# 肺癌驱动基因与PD-1/PD-L1信号通路相互作用在非小细胞肺癌发生发展中的研究进展

**DOI:** 10.3779/j.issn.1009-3419.2017.11.10

**Published:** 2017-11-20

**Authors:** 岩 石, 望 吕, 路明 汪, 坚 胡

**Affiliations:** 310003 杭州，浙江大学附属第一医院胸外科 Department of Thoracic Surgery, the First Affiliated Hospital, Zhejiang University, Hangzhou 310003, China

**Keywords:** 程序性死亡分子1, 程序性死亡分子1配体, 肺肿瘤, 肺癌驱动基因, Programmed death 1, Programmed death 1 ligand, Lung neoplasms, Lung tumor driver gene

## Abstract

程序性死亡分子1（programmed death 1, PD-1）/程序性死亡分子1配体（programmed death 1 ligand, PD-L1）通路是免疫调节的重要通路，而这一通路在肿瘤组织中存在着异常激活，提示PD-1/PD-L1通路可能参与了肿瘤的免疫逃逸过程。肿瘤驱动基因在非小细胞肺癌（non-small cell lung cancer, NSCLC）的发生发展中发挥着重要的作用，而对于肿瘤免疫逃逸的建立同样具有潜在的作用，这提示肿瘤驱动基因通路与PD-1/PD-L1通路可能存在相互作用。本文将对目前关于PD-L1与主要的肺癌驱动基因表皮生长因子受体基因（epidermal growth factor receptor, *EGFR*）、鼠类肉瘤病毒癌基因（Kirsten rate sarcoma viral oncogene homolog, *KRAS*）及棘皮微管样蛋白4-间变性淋巴瘤激酶融合基因（echinoderm microtubuleassociated protein-like 4 -anaplastic lymphoma kinase, *EML4-ALK*）之间的关系及调控进行综述，总结肺癌驱动基因及PD-1/PD-L1通路相互作用在非小细胞肺癌发生发展中的作用。

## Abstract (en)

Programmed death 1 (PD-1) and programmed death 1 ligand (PD-L1) pathway is a key mechanism of immune regulation, and its abnormal activation in tumor tissues suggests that PD-1/PD-L1 pathway may participate in the regulation of tumor immune escape. Driver gene mutation which is known as a key factor in the tumorigenesis of non-small cell lung cancer (NSCLC), was also reported to play a important role in the process of tumor immune escape. It indicates that there is an interaction between driver gene and PD-1/PD-L1 pathway. The purpose of this paper is to review the relationship between PD-1/PD-L1 pathway and lung cancer driver gene, such as epidermal growth factor receptor (EGFR), Kirsten rate sarcoma viral oncogene homolog (KRAS) and echinoderm microtubuleassociated protein-like 4 -anaplastic lymphoma kinase (EML4-ALK) and to summarize the role of lung cancer driver gene and PD-1/PD-L1 pathway interaction in the tumorigenesis and progression of NSCLC.

肺癌是目前世界范围内发病率和死亡率最高的恶性肿瘤之一^[1]^，肺癌病例占到所有肿瘤新发病例的13%，死亡病例则占到所有肿瘤死亡病例的18%^[2]^。非小细胞肺癌（non-small cell lung cancer, NSCLC）是肺癌中最为常见的组织类型，占到所有类型肺癌的80%-85%^[1]^，其中70%NSCLC病例为腺癌或鳞癌^[3]^。中国是肺癌高发国家，肺癌的发病率和死亡率均居世界首位^[4]^，且发病率逐年上升，肺癌的防治工作面临严峻挑战。

免疫系统与肿瘤的发生发展存在着密切的联系，一方面免疫系统能够通过多种免疫效应机制杀伤或清除肿瘤细胞，另一方面肿瘤细胞也能够通过多种免疫逃逸机制抵抗或避免疫系统对肿瘤细胞的杀伤或清楚。在肿瘤免疫中，免疫检查点分子（immune checkpoint）是肿瘤基础和临床研究中的热点问题。T淋巴细胞介导的免疫反应是机体抗肿瘤免疫的中心环节，T淋巴细胞作用的强度和效应始终处于多种共刺激信号和共抑制信号的精确调控下，参与T淋巴细胞共抑制信号的分子，称之为免疫检查点分子，如细胞毒性T细胞相关抗原4（cytotoxic T lymphocyte-associated antigen-4, CTLA-4）、细胞程序性死亡分子1（programmed death 1, PD-1）及其配体（programmed death 1 ligand, PD-L1）等^[5]^。免疫检查点对于实现免疫反应的精准控制具有关键作用，肿瘤细胞介导的免疫检查点的异常活化是肿瘤细胞免疫逃逸的重要途径之一。这也为肿瘤的靶向治疗提供了新的靶点，CTLA-4、PD-1/PD-L1被认为是目前最有效的治疗靶点^[6]^。

在NSCLC中，肿瘤驱动基因（tumor driver gene）突变引起的信号通路异常激活是肿瘤发生的重要机制，已有研究表明肿瘤驱动基因突变与PD-1/PD-L1信号的异常激活存在着密切的联系，从而介导了NSCLC的发生与发展。同时也提示了针对肿瘤驱动基因靶向治疗与PD-1/PD-L1阻断治疗联合应用的可能。

## 1 肺癌驱动基因

基于肿瘤基因水平的研究，已经确定了许多与肿瘤诱发和维持恶性肿瘤的分子改变，称为肿瘤驱动基因。肿瘤驱动基因改变是NSCLC发病的重要机制之一，尤其是在肺腺癌中，功能性驱动基因的突变率约为60%，其中表皮生长因子受体（epidermal growth factor receptor, *EGFR*）、鼠类肉瘤病毒癌基因（Kirsten rate sarcoma viral oncogene homolog, *KRAS*）以及棘皮微管样蛋白4（echinoderm microtubule associated protein-like 4, *EML4*）-间变性淋巴瘤激酶（anaplastic lymphoma kinase, *ALK*）融合基因（*EML4-ALK*）最为常见，占35%-40%^[7]^。*EGFR*是肺腺癌中最重要的驱动基因，EGFR为酪氨酸跨膜受体，在NSCLC中外显子19缺失突变（delE746-A750）及858位亮氨酸替代精氨酸突变（L858R）是最常见的突变方式^[8]^。*EGFR*突变后能够导致其自磷酸化，激活酪氨酸激酶，并进一步激活RAS/RAF/MEK/MAPK通路、PI3K/AKT/mTOR通路，参与肿瘤细胞的增殖、生长、侵袭、转移及血管生成^[9]^。*KRAS*基因属于*RAS*基因家族，为EGFR信号下游重要的调节位点，参与细胞增殖与分化的调节，其主要突变位点发生在12、13和61密码子，其中密码子12突变占到90%^[10]^，突变使得在缺乏生长因子信号的条件下也可激活RAS-GTP酶，使得细胞获得持续增殖的能力。*EML4-ALK*融合基因是由2号染色体EML4在内含子13位置处断裂与*ALK*基因19内含子位置连接形成，*EML4-ALK*融合基因能够异常激活下游MAPK、PI3K、STAT等信号通路，介导细胞增殖与侵袭，并且参与细胞凋亡的抑制^[11]^。

基于驱动基因，特别是EGFR信号通路的靶向治疗药物已经逐渐成为NSCLC治疗的趋势，吉非替尼（Gefitinib）和厄洛替尼（Erlotinib）等表皮生长因子酪氨酸激酶抑制剂（EGFR tyrosine kinase inhibitor, EGFR-TKI）已经在NSCLC晚期患者中得到成功应用，使得患者的无疾病进展生存期（progression-free survival, PFS）和总生存期（overall survival, OS）均得到显著延长^[12, 13]^。

## 2 PD-1/PD-L1通路

PD-1属于免疫球蛋白B7-CD28家族，为Ⅰ型跨膜蛋白^[14]^，主要表达于活化的免疫细胞，包括T细胞、B细胞、NKT（natural killer T）细胞、树突状细胞、单核-巨噬细胞等^[15]^，由于PD-1分子具有免疫受体酪氨酸抑制基序（immunoreceptor tyrosine-based inhibitory motif, ITIM）、免疫受体酪氨酸转换基序（immunoreceptor tyrosine-based switch motif, ITSM），为免疫抑制性受体，T细胞PD-1表达是T细胞应答疲劳的重要标志^[14]^。

PD-L1（B7-H1）为PD-1的主要配体，主要表达于T细胞、B细胞、树突状细胞、肥大细胞等造血细胞来源细胞^[16]^，在内皮细胞和多种上皮细胞中也有表达^[17]^。而在NSCLC、黑色素瘤、乳腺癌、胶质瘤等多种恶性肿瘤中，PD-L1也有高表达，提示这可能是肿瘤免疫逃逸的重要机制。研究表明，PD-1与PD-L1的相互作用对CD4^+^及CD8^+^ T细胞的增殖分化起到抑制作用，降低多种免疫因子的表达，并促进活化的效应细胞毒性T细胞（cytotoxic lymphocyte, CTL）凋亡，介导肿瘤细胞逃避CTL的杀伤^[18]^。除此之外，PD-L1还能够增强调节性T细胞（regulatory T cells, Tregs）的免疫抑制作用^[19]^。这表明，PD-1/PD-L1通路通过抑制肿瘤局部微环境的免疫效应，参与肿瘤的免疫逃逸，促进肿瘤的生长和转移。

在NSCLC病例中，19.63%-65.38%的病例发现有PD-L1的高表达^[20-24]^，其中女性患者、非吸烟病例中PD-L1高表达更为明显，并且腺癌病例中PD-L1表达显著高于鳞癌^[20]^，这提示肿瘤发生机制的差异与PD-L1的表达相关。PD-L1的高表达提示患者预后较差^[20, 25]^。这都表明PD-1/PD-L1通路是NSCLC发生发展中的一个关键分子途径。目前针对PD-1/PD-L1通路的免疫靶向药物在NSCLC治疗中已经表现出令人惊喜的效果，纳武单抗（Nivolumab, Opdivo）、派姆单抗（Pembrolizumab, Keytruda）已经相继被美国食品药品管理局（Food and Drug Administration, FDA）相继批准用于NSCLC的治疗。在多项临床试验中，PD-1免疫靶向治疗患者的缓解率（overall response rate, ORR）、5年生存率、OS均有了显著的提高，取得了优于传统化疗药物的治疗效果^[26-28]^。需要引起注意的是肿瘤PD-L1水平可能是免疫靶向治疗疗效的一个重要影响因素，PD-L1阳性患者的ORR显著高于PD-L1阴性患者^[27, 28]^。这均提示PD-1/PD-L1通路在肿瘤发生发展中的重要作用，但PD-1/PD-L1通路在肿瘤组织中激活与调控机制尚未完全阐明，肿瘤驱动基因在PD-1/PD-L1的调控中作用机制已成为前沿热点。

## 3 PD-L1表达与肺癌驱动基因EGFR的关系及调控

*EGFR*基因是NSCLC中最重要的驱动基因之一。目前已经有研究反映了EGFR与PD-L1表达之间存在一定的联系。在亚洲NSCLC患者中携带有*EGFR*突变的比例占到47.9%^[29]^，并且在女性、非吸烟及腺癌病例中，*EGFR*突变较高^[30]^，这与PD-L1的表达模式相一致。Azuma等^[20]^对手术切除的NSCLC肿瘤样本进行的分析表明*EGFR*突变与PD-L1表达之间存在着相互关系。在人NSCLC细胞系中，携带有*EGFR*突变的细胞中PD-L1的表达要显著高于EGFR野生型细胞^[6, 20]^。Akbay等^[24]^发现激活EGFR通路，PD-L1的表达上调，并通过PD-1/PD-L1通路的激活介导了肿瘤的免疫逃逸。研究^[6, 20, 24, 31]^还发现，EGFR-TKI能够抑制TKI敏感细胞系中EGFR信号的活化，降低PD-L1的表达，但在EGFR-TKI抗性的NSCLC细胞系中，EGFR-TKI则不能够影响PD-L1的表达水平。但也有研究^[31]^却得出了相反的结论，发现在EGFR-TKI抗性细胞系中，PD-L1表达水平要高于EGFR-TKI敏感细胞系，EGFR-TKI同样能够降低抗性细胞系中PD-L1的表达。这都表明PD-L1表达与EGFR信号通路有着密切的联系，PD-L1的表达受到EGFR信号的调控。Chen等^[6]^认为，EGFR对PD-L1的调控是通过ERK1/2/c-Jun通路实现的，*EGFR*基因突变会使得EGFR磷酸化水平提高，激活EGFR通路，磷酸化ERK及c-Jun，从而上调PD-L1的表达水平，而AKT通路在这个过程中并不发挥作用。但也有研究^[32]^表明，PI3K-AKT通路与MEK-ERK通路均参与了EGFR对PD-L1的调控，利用PI3K-AKT通路及MEK-ERK通路的特异性阻断剂，均能够观察到PD-L1表达水平的显著下调。NF-κB被认为是PD-L1在单核细胞中表达重要调控因子，它能够直接结合于PD-L1的启动子，直接参与PD-L1表达调控^[33]^，Lin等^[31]^则发现在NSCLC细胞中，NF-κB仍在PD-L1的调控中发挥作用，EGFR-TKI能够通过抑制NSCLC细胞中NF-κB的表达，当NF-κB表达下调后，PD-L1的表达水平也随之下调，这提示EGFR上游信号激活后，NF-κB可能作为直接的转录调控原件参与了PD-L1表达的调控。

EGFR与PD-L1之间的关系还存在有很大的争议，也有很多研究表明，PD-L1的表达与EGFR通路并无相关性，Zhang等^[34]^检测了143份手术切除的肺癌组织标本，在*EGFR*突变型肺癌组织中发现PD-L1和PD-L2表达阳性与阴性的例数相比并无显著性差距。因此还需要更加深入的研究，以明确EGFR同PD-L1表达之间的关系及具体机制（图 1）。

**1:**
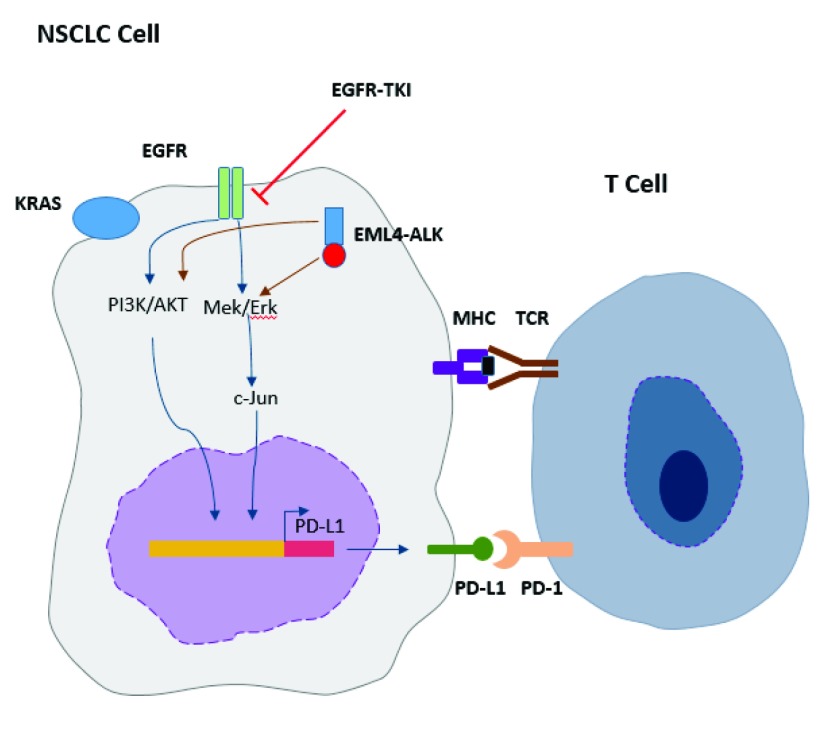
PD-L1表达与EGFR信号通路关系 The relationship between PD-L1 expression and EGFR pathway. NSCLC: non-small cell lung cancer; EGFR: epidermal growth factor receptor; PD-L1: programmed death 1 ligand.

在EGFR-TKI的治疗过程中，EGFR-TKI耐药性的产生严重限制了患者在EGFR-TKI靶向治疗中的收益。由于*EGFR*突变和PD-L1表达之前可能存在着一定相互关系，EGFR-TKI与PD-1单抗的联合应用，可能对提高患者治疗效果提供了新思路。2014年第50届美国临床肿瘤学会（American Society of Clinical Oncology, ASCO）年会上，公布了Nivolumab联合厄洛替尼治疗EGFR-TKI继发耐药NSCLC患者的临床试验，结果显示Nivolumab联合厄洛替尼可带来持续性临床获益，其ORR为19%。这也提示PD-1单抗与EGFR-TKI联合治疗可显著提高NSCLC患者的治疗效果（图 2）。

**2:**
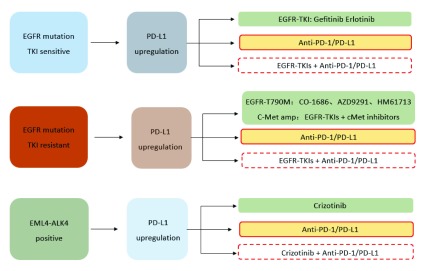
NSCLC靶向治疗与PD-1/PD-L1免疫治疗 Targeted therapy and PD-1/PD-L1 targeted immune therapy in NSCLC. PD-1: programmed death 1.

## 4 PD-L1表达与肺癌驱动基因*EML4-ALK*间的关系及调控

*EML4-ALK*也是NSCLC重要的驱动基因，中国NSCLC患者中的阳性率为3%-11%^[35-37]^，多见于年轻、不吸烟或少量吸烟患者及EFGR和KRAS阴性的肺腺癌患者^[38]^。有研究^[20]^发现，EML4-ALK阳性细胞的PD-L1表达水平显著高于EML4-ALK阴性的细胞，并且EML4-ALK融合蛋白的表达能够上调PD-L1的表达，抑制ALK的活性或敲除EML4-ALK后，同样能够降低PD-L1的表达。这表明PD-L1的表达受到EML4-ALK融合蛋白的调控。进一步的研究表明，MEK-ERK及PI3K-AKT通路参与了EML4-ALK调控PD-L1表达的过程，这一信号通路与EGFR的调控途径相似，表明*EML4-ALK*与*EGFR*这两个不同的癌基因可能共享相同的调控通路来调控PD-L1的表达。

## 5 PD-L1表达与肺癌驱动基因*KRAS*间的关系及调控

15%-20%的NSCLC患者携带有*KRAS*基因突变，而且常发生在吸烟的肺腺癌患者中^[37]^。KRAS与PD-L1表达之间的关系目前研究较少，仍存在一定的争议。D’Incecco等^[39]^的研究观察到，PD-L1的表达与*KRAS*基因突变存在显著的相关性（*P*=0.006）。但也有很多研究持有不同的观点，Calles等^[40]^研究了*KRAS*突变的NSCLC患者中PD-L1的表达水平，发现在*KRAS*突变的NSCLC患者中，有24%表现为PD-L1阳性，*KRAS*的主要突变类型*KRAS* G12C、*KRAS* G12D、*KRAS* G12V、*KRAS* G13X及KRAS Q61H均与PD-L1的表达水平无显著相关性。KRAS信号可能不参与PD-L1表达的调控^[24, 36]^。

## 6 展望

PD-1/PD-L1信号通路与肺癌驱动基因存在着一定的相互作用，特别是在EGFR可能通过PI3K-AKT通路与MEK-ERK通路上调PD-L1的表达，从而介导肿瘤免疫逃逸。肺癌驱动基因与免疫检查点分子间的相互作用对于肿瘤的发生与发展具有重要的意义。但两者间的调控机制尚未完全阐明，*EGFR*、*KRAS*突变与PD-L1表达水平间的关系仍存在着争议，表明肺癌中PD-1/PD-L1的调控是一个复杂的过程，可能涉及到多个驱动基因及多条信号通路，需要进一步的研究和探讨。但目前，基于PD-1/PD-L1的免疫靶向治疗已经在NSCLC的治疗中取得了良好的效果，对解决EGFR-TKI耐药性的制约提供了新的方法^[41]^，EGFR-TKI联合阻断PD-1/PD-L1的治疗模式为提高肺癌患者长期生存带来了新的希望。随着对PD-1/PD-L1及肺癌驱动基因研究的深入，将丰富我们对肿瘤发生、发展及免疫逃逸机制的认识，为肿瘤的精准治疗提供新的方向。
